# Tobacco Smoking and Use of Smokeless Tobacco and Their Association with Psychological Distress and Other Factors in a Rural District in Bangladesh: A Cross-Sectional Study

**DOI:** 10.1155/2019/1424592

**Published:** 2019-12-06

**Authors:** Fakir M. Amirul Islam, Alexandra Walton

**Affiliations:** ^1^Department of Statistics, Data Science and Epidemiology, Faculty of Health, Arts and Design, Swinburne University of Technology, Melbourne, Australia; ^2^Organisation for Rural Community Development (ORCD), Dariapur, Narail, Bangladesh

## Abstract

**Background:**

Tobacco smoking and use of smokeless tobacco are the most preventable cause of death in Bangladesh. The prevalence of psychological distress is increasing globally. This paper reports the smoking status and their association with psychological distress and other factors in a rural district, Narail, of Bangladesh.

**Materials and Methods:**

Data were collected from 2425 adults of age 18–90 years. Smoking status along with sociodemographic characteristics and measures of psychological distress using the Kessler 10-item questionnaire were collected using a face-to-face data collection method.

**Results:**

The crude (age-standardized) prevalence of ever smoking was 27.1 (24.3)% that includes current 25.6 (23.7) and smoker 1.5 (0.6)%, and the prevalence of smokeless tobacco (SLT) was 23.5 (13.4)%. The prevalence of ever smoking was the highest in daily labourers (62.9%) and SLT use was the highest in widowed people (47.2%). After adjustment for covariates, no education (odds ratio (OR): 3.78, 95% confidence interval (CI): 1.57–9.07 for females and OR: 2.69, 95% CI: 1.87–3.87 for males) compared to at least secondary level of education and daily labours (OR: 6.66, 95% CI: 1.67–26.6 for females and OR: 5.12, 95% CI: 1.30–20.19 for males) compared to housework were associated with higher prevalence of ever smoking. Any level of psychological distress, such as mild psychological distress, was associated with at least double the prevalence of tobacco smoking in females (OR: 2.12, 95% CI: 1.67–3.83) but not in males (OR: 1.12, 95% CI: 0.80–1.56). Psychological distress was not associated with SLT use.

**Conclusions:**

Prevalence of both smoking and SLT use was high, particularly in daily labourers, people with no education, and females with psychological distress in rural Bangladesh. Appropriate intervention programs should especially target those of low levels of education and laborious occupations for increasing awareness for the cessation of smoking in rural Bangladesh.

## 1. Introduction

According to the World Health Organisation's global report, 1.1 billion people smoke tobacco, 80% of them live in low- and middle-income countries, and 8 million people die from tobacco smoking [1]. Smokeless tobacco (SLT) can cause serious health problems including cancers [2] and cardiovascular diseases (CVD) [3–7]. The prevalence of both tobacco smoking and SLT use is consistently more common among males than females across the globe [8–11], except for some African and Asian countries, including Bangladesh, where SLT use is more prevalent among females [9, 12].

Bangladesh is a densely populated country, with a total population of 163 million people in approximately 1.2 million square kilometres of land [13]. Smoking is one of the biggest preventable causes of death in Bangladesh, with a major public health burden of morbidity, disability, mortality, and community costs [14, 15]. A study conducted by Alam et al. [16] suggested that 25% of all deaths among males aged 25–69 years were attributable to smoking which leads to an average loss of 7 years of life per smoker. According to a nationwide Global Adult Tobacco Survey (GATS), in 2009 for the age of 15 years and above [14], the overall prevalence of smoking was 23% and SLT use was 27.2% and the prevalence of smoking was higher in males and SLT use was higher in females. Sreeramareddy et al. [17] reported that the prevalence of smoking was 60% and SLT use was 21.35% in males of age between 15 and 64 years from another national representative survey. The Non-Communicable Disease (NCD) Risk Factor Survey 2010 for the age of 25 years and older observed an overall prevalence of smoking (26.2%) that was also significantly higher in males [18]. The difference in the prevalence of smoking among all these national representative samples can be attributed to that the smoking is more prevalent among the middle-aged people and the lower prevalence of SLT use is associated with younger people [9, 19, 20]. Thus, a wide range of age distribution is required to report more robust data on smoking.

Smokeless tobacco has been associated with the development of many diseases including oral cancer [21] and cardiovascular diseases [5, 22] although evidence exists that it was not associated with cardiovascular diseases [23]. Up until 2013, there had been no policy of cessation of SLT use in Bangladesh [5]. Moreover, its use, especially by women, was associated with positive attitudes [20]. The cessation of SLT use was also found to be associated with a significant increase in anxiety and oral pain [24]. Therefore, the effect of SLT use could be studied using longitudinal follow-up study. The prevalence of psychological distress is quite high [25] and a serious cause of morbidity and mortality in Bangladesh [26, 27], which is reported to be associated with smoking in some countries such as in the United States [28] and Australia [29]. To our knowledge, the association of psychological distress with smoking or SLT use has not been explored in Bangladesh, and thus, it is unknown if the association of psychological distress and smoking in Bangladesh is similar or different from that found in the United States or Australia. In Bangladesh, there is a social inequality between males and females [30]; for example, females are financially more dependent and have comparatively low levels of education [31], and have low engagement in different occupations compared with males [32]. Thus, combined analyses that were usually performed previously potentially mask important differences in factors associated with smoking between males and females and require to explore a separate analysis for males and females. The current study aimed to report both smoking tobacco and SLT use and the factors including psychological distress and major occupations in association with smoking status for males and females in a large sample from a rural district of Bangladesh.

## 2. Materials and Methods

### 2.1. Study Sample

The current cross-sectional study recruited 2425 participants of age between 18 and 90 years from the Narail district of Bangladesh. The description of Narail district and its location map was described elsewhere [13]. The sample size was calculated based on the prevalence of depression and anxiety which has been described in detail previously [33]. For this current study, the sample size of 2425 was 98% powered at a significance level of 0.05 to show a prevalence of current smoking of 23% [14] with a 95% confidence interval.

### 2.2. Recruitment and Methods of Data Collection

We used a multistage cluster random sampling technique for data collection. Participants from Narail Upazila originated from 13 rural unions and 9 wards under the urban city of Narail. Bangladesh is divided into 8 main administrative divisions, each of which is divided into a number of districts, and thus there are 64 districts, or Zila. Each district is composed of a number of Upazilas, each of which is again divided into some rural unions and an urban pourashava. A rural union has a number of villages with 200–400 households, and an urban pourashava is subdivided into a number of wards, which again divides into a number mahallas that comprised approximately 500 households. Narail is such an Upazila. Three unions from a total of 13 rural unions and one ward from a total of 9 urban wards of Narail were randomly selected at level 1. Two or three villages or mahallas from each of the unions or wards were randomly selected at level 2. Approximately 120 older adults and 150 adults were interviewed from each village. Recruitment strategy and quality assurance in data collection were described in detail previously [33]. Data were collected by three teams, each of which had 3 members. An interviewer-administered semistructured questionnaire was developed to collect relevant sociodemographic data including participants' levels of education and SES. The well-established Kessler 10-item (K10) questionnaire was used for measuring psychological distress [34].

The interviewers were instructed to visit every household within the randomly selected villages and to interview one member from each household. If there were more than one male or female adult in the same household, one individual was selected, based on whose date of birth was closer to January. However, to maintain an approximately equal number of male and female participants, one female was interviewed immediately after a male participant. The recruitment started from a corner of a village and continued until the recruitment of a maximum of 250 participants was reached for a large village if the number of eligible participants was greater than 250. In the case of fewer than 250 households in a village, the recruitment continued to the adjacent village to reach the number 250. We have reported the study sample, recruitment strategy, and data collection techniques in previous studies [33, 35].

### 2.3. Outcome Variables

The outcome variable of the current study was smoking status, and it was defined into three categories: “Never smoking” was defined as participants who had never smoked throughout their life. “Ever smoking” was classified as any participant who was a previous tobacco smoker or a current tobacco smoker, whether daily or occasional. Ex-smoker was classified as if someone smoked previously but had quit completely at least three months ago. “Tobacco smoke” was defined as participants who smoked any type of bidi or cigarette. “Smokeless tobacco” was defined as participants who currently consumed any type of smokeless tobacco product, such as “gul,” which is ash from the tobacco leaf that is sniffed and/or stuck on their teeth, sun-dried or cured raw leaf, which is known as “sada patha” that is chewed with betel leaf, and “zarda,” which is commercially manufactured from tobacco leaf, lime, and species that is also chewed with betel leaf. The people were categorised as smokers if they consumed tobacco mainly and SLT occasionally. Similarly, people were categorised as SLT users if they mainly consumed SLT but smoked tobacco occasionally. However, if people consumed tobacco or SLT with a similar proportion, they were considered to be both smokers and SLT users.

### 2.4. Independent Variables

Demographic details for age, gender, and level of education, which is categorised into no schooling, primary school level of education (grades 1 to 5), secondary school level of education (grades 6 to 10), and school secondary certificate (SSC) or above, were collected. SES was assessed according to Cheng et al.'s study [36] asking whether “over the last twelve months, in terms of household food consumption, how would you classify your socioeconomic status,” with the possible answers as follows: (i) insufficient funds for the whole year, (ii) insufficient funds some of the time, (iii) neither deficit nor surplus (balance), and (iv) sufficient funds most of the time. Data on current occupation (e.g., student, housework, farming, daily labours, business, government or nongovernment job, and retired or unable to work), marital status (married, widowed, never married, or single), current health problems such as diabetes and high blood pressure (yes or no), and number of health problems were also collected during the interview. The data related to health problems were self-reported. Psychological distress was defined based on the sum of five-value response options for each of the K10 questionnaire [34] with a maximum score of 50 and the minimum score of 10. The scores were categorised as no psychological distress (10 to 19) and mild (20 to 24), moderate (25 to 29), and severe psychological distress (30 to 50) as per Andrews and Slade [37] and Kessler et al. [34].

### 2.5. Statistical Analysis

Participant's age, gender, level of education, marital status, SES, occupation, existence, and a number of health conditions were reported using descriptive statistics by gender. Smoking status was examined in association with each of the sociodemographic characteristics using Chi-square tests for the total sample, as well as separate analyses were performed for males and females. The age-standardized prevalence for “current,” “ex-smoker,” “ever smoker,” and “SLT use” was calculated using the population size of different age groups at a national level. We used a direct standardized method [∑(*r*_*i*_ × *P*_*i*_)/∑*P*_*i*_] to calculate age-standardized prevalence. Here, *r*_*i*_ is assumed to be the prevalence of smoking and SLT use in age group *i* and *P*_*i*_ is the population size in the *i*th age group [38]. We used binary logistic regression models to compute odds ratio (OR) and 95% confidence intervals (CI) for ever-smoker and use of SLT in association with sociodemographic and psychological factors for the total sample as well as for males and females separately. The covariates for adjustment were age, gender and level of education, occupation, and marital status for the total sample. However, for separate analyses for males and females, the covariates were age, level of education, occupation, and marital status as these are reported to be significantly associated with smoking status [19]. Although SES has been reported to be associated with smoking status, SES is positively correlated with the level of education and thus both are not used for adjustment of the same model to avoid overadjustment problems. Since the number of ex-smokers is small, the models were fitted for ever smokers throughout the manuscript. The statistical software was used SPSS (SPSS Inc, version 21).

## 3. Results

The mean (SD) age of the total participants was 52 (17) years. Of the total participants, 51% were females. Percentages of no education (31% in females vs. 24% in males) (*p* < 0.001) and widowed (27% in females vs. 6.1% in males) (*p* < 0.001) were higher in females. The females were more occupied in house duties (77.5% in females vs. 1.4% in males) and less occupied in laborious work (0.8% in females vs. 22.9% in males were daily labourers) and in business (0.2% in females vs. 18.4% in males) (*p* < 0.001). Proportions of females and males by psychological distress in the total sample were similar (*p*=0.19) (Table 1).

### 3.1. Smoking Status by Age and Gender

In the total sample, the prevalence of current smoker was 25.6%, ex-smoker was 1.5%, SLT user was 23.5%, and both tobacco and SLT user was 0.8%. Of 36 (1.5%) ex-smokers, all were male and 94% of them were older than 60 years. All ex-smokers had at least one medical condition, and all were on medications. Of 19 (0.8%) participants who smoked both tobacco and SLT, all were male and 18 were older than 40 years (Table 2).

### 3.2. Prevalence of Smoking and SLT Use in Lifespan

**Figure 1:**
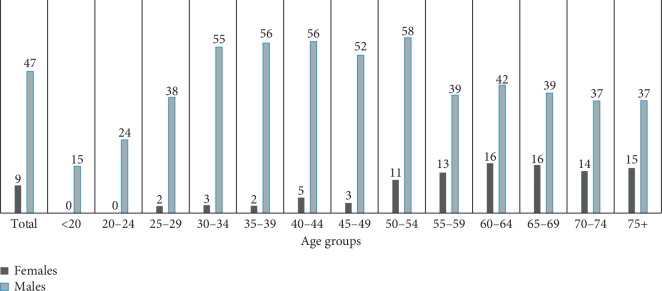
Percentage of ever smoking by age group for females and males.

**Figure 2:**
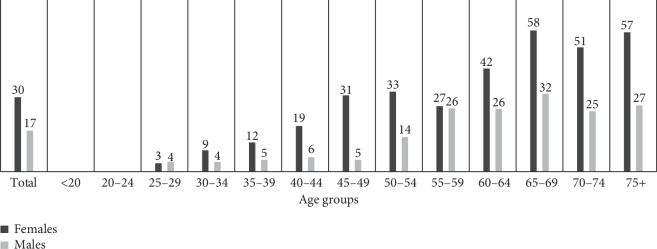
Percentage of smokeless tobacco by age group for females and males.

The prevalence of smoking was higher among males than females at every point in the lifespan. In females, ever smoking was most prevalent in older ages with a slight increase at the age of 55, which remained constant after this age. In males, ever smoking was most prevalent within the age range of 30 to 55 years and a decline was observed after this age (Figure 1). The prevalence of SLT use was higher among females than males at almost every point in the lifespan (Figure 2).

### 3.3. Factors Associated with Tobacco Smoker and SLT User in the Total Sample

In the total sample, the prevalence of ever smoking was 27.1%. The highest prevalence was among daily labourers (62.9%), followed by the landowners (57.5%), people with a business occupation (50.2%), people having insufficient funds all the time (37.1%), and people with severe psychological distress (33.0%). After adjustment for age and level of education, male sex (odds ratio (OR) 8.38; 95% confidence interval (CI): 6.63–10.6) was associated with higher prevalence of ever smoking but there was no significant difference between males and females in SLT consumption (OR 0.91; 95% CI: 0.74–1.12) (Table 3).

### 3.4. Factors Associated with Tobacco Smoker by Gender

Irrespective of gender, no or primary level of education, laborious occupations, and having any health condition were associated with a higher prevalence of tobacco smoking. In females only, experiencing any level of psychological distress was associated with a higher prevalence of tobacco smoking (in females with and without psychological distress, the smokers were 19.0% vs. 4.6%). After adjustment for confounding factors, older age, no education or primary level of education, and laborious work such as landowners and daily labourers were associated with a higher prevalence of ever smoking both in females and males. For example, the occupation of daily labour was associated with the highest prevalence of ever smoking in both females (54%, OR 6.62, 95% CI 1.67–27.0) and males (61%, OR 5.12, 95% CI 1.3–20.2). Any level of psychological distress was associated with a higher prevalence of ever smoking only in females, such as mild psychological distress (OR 2.12; 95% CI 1.67–3.83) (Table 4, columns 4–7).

### 3.5. Factors Associated with SLT User by Gender

The prevalence of SLT use between the age of 60 and above was high, 46.6% in females and 27.1% in males compared to that in the younger age group of 18–39 years, which was 7% in females and 3.7% in males.

Prevalence of SLT use was high in people with no or primary education (OR: 4.4; 95% CI: 2.82–6.9 for females and OR: 2.64; 95% CI 1.62–4.28 for males) compared to secondary or above education and people who had any health conditions (OR: 1.86; 95% CI: 1.14–2.6 for females and OR: 1.80; 95% CI 1.16–2.78 for males) compared to those who did not have any health conditions. Older age was also associated with a higher prevalence of SLT use. Other factors including the psychological distress were not significantly associated with a higher prevalence of SLT use (Table 4, columns 8–11).

## 4. Discussion

Our study compared the likelihood of smoking and SLT use through a range of sociodemographic factors for both males and females in a large sample over a wide age distribution. Our study also reports the association of psychological distress measured by the internationally validated K10 psychological distress tool with smoking. The prevalence of tobacco smoking was more prevalent in males than females. SLT use was more prevalent in females than in males. These observations are similar to the findings reported by the GATS [14] and some other studies in Bangladesh [12, 20]. This finding further contributes to a large body of evidence demonstrated in previous studies [8–12, 39]. Tobacco smoking was generally more prevalent in middle age and above, while SLT use was more prevalent as age increased after 40 years. Low levels of education and laborious occupation were significantly associated with tobacco smoking in both sexes. Psychological distress was found to be associated with a higher prevalence of smoking in females only.

Smoking has enormous consequences not only on health but also on the economy and wellbeing. In agreement with previous reports, we found that the prevalence of tobacco smoking was higher in males compared to females [12, 20]. Importantly, we noted that while the prevalence of tobacco smoking is decreasing in males, it is not diminished in females [40]. The GATS found that 44.7% of males compared to 1.5% of females aged 15 years or older were smokers [14]. This indicated that the prevalence of smoking in males in this typical rural community is either stable or declining over time but the prevalence of smoking in females remains alarmingly high. Female smoking has serious consequences on their health including increased risk for breast and cervical cancer and cardiovascular disease development, as well as exerting a negative impact on economic sufficiency and wellbeing [41].

The observation of an association of lower prevalence of smoking with better SES for both males and females in our study supports the previous findings found in Bangladesh, India, Thailand, and Indonesia [9]. Thus, our data strengthen evidence about the negative relationship between these two variables. However, our study demonstrates and adds to the literature that irrespective of gender, the prevalence of smoking is higher in people with laborious occupations, which is consistent with some previous studies [42, 43], and that psychological distress is associated with smoking tobacco in females.

The similar prevalence of SLT use in Bangladesh and India may be due to both countries having similar socioeconomic conditions and a shared cultural heritage [44]. The association of older age with a higher prevalence of smoking and SLT use can be explained as the lower level of education, which is strongly associated with older age [45], is one of the main driving forces of ignorance that influences the higher prevalence of smoking status. Especially in females, tobacco smoking was more prevalent among the age over 60 years, where 75.0% did not have any formal education. In comparison, the prevalence of smoking in males was highest among the age range of 39 to 54 years. The higher prevalence within this age range can be attributed to the fact that most of the people are in the workforce, including daily labourers or landowners, who were found to be associated with the higher prevalence of tobacco smoking. The lower prevalence of smoking later in life is possible that poor health, which is common during the later stages of the lifespan, acts as a motivation to cease smoking or to switch to other forms of tobacco consumption, such as SLT use, which would simultaneously account for the decrease in smoking prevalence of men in later stages of life and the increase of SLT use in older age. In our study, we found that 1.5% of the total or 3% of the men were ex-smoker, of whom all had at least one medical condition and were on medication. These findings support the fact that many elderly people who smoke tobacco are more likely to be successful at quitting smoking, especially when they have other health problems [46].

The association of psychological distress with smoking only in females can be attributed to that the females are more exposed to lower levels of education, physical ill health, and family violence which are the common risk factors of psychological distress and leads females to take up smoking [47]. Societal values impact smoking and SLT use prevalence worldwide [48]. Among South-East Asian countries, including Bangladesh, it is considered taboo for women to smoke tobacco; however, this restriction does not apply to SLT products, which have become an integral part of Bangladeshi tradition, particularly for women. Common cultural celebrations which include SLT products include marriage, entertainment, festivals, and rituals [44, 49]. Moreover, culturally, women are criticised for smoking tobacco in public; however, public consumption of SLT by women is not considered to be improper, which likely contributes to higher consumption of SLT use in females [49]. Attitudes towards tobacco consumption are considered to be risk factors for health, and SLT use is considered to be less harmful than smoking tobacco and that increases the likelihood of using the SLT [9, 20].

Strengths of our study: Firstly, the face-to-face data collection from a large sample with almost 50% females from a rural district in Bangladesh. Secondly, data were collected from adults and older adults with a wide age distribution covering the whole spectrum of adults. Thirdly, analyses were performed for total samples as well as for males and females separately to obtain an in-depth understanding of the risk factors of smoking and SLT use given the fact that the prevalence of smoking is different in males and females. This is the first study in Bangladesh to report the associations of psychological distress measured using the Kessler 10-item questionnaire with smoking tobacco and the use of SLT. The K10 tool was validated for its psychometric properties to use in rural Bangladesh [35]. Although there are some other tools for measuring depression and anxiety, such as the Hospital Anxiety and Depression Scale (HADS) [50] and the Depression Anxiety Stress Scale (DASS) [35, 51], none of these were validated for their psychometric properties to measure psychological distress or depression or anxiety in general people in rural Bangladesh.

Limitations include, firstly, the collection of data from one district, and thus, the results may not be generalised at the national level. Whilst it is representative of the situation in Narail district, and the rural population is very homogenous in Bangladesh, the study's results need to be extrapolated with caution to other rural parts of Bangladesh. Secondly, data on smoking and SLT use were based on self-report and the duration of smoking or the pack-years of smoking was not collected to confirm if there was any inconsistency in self-reported data. Thirdly, data on health conditions were self-reported. Reporting bias, reporting errors, and different perceptions about their health conditions are very likely dependent on disease severity and the level of knowledge of the participants. Therefore, the association of any medical conditions with smoking status needs to be reported with caution. We had not collected data on SLT in the past. Finally, although we have validated the psychometric properties of the Kessler 10-item questionnaire and proposed seven items, we could not identify the new cutoff to define the psychological distress yet.

## 5. Conclusions

Our study succeeded in providing further information which is useful when it comes to understanding how sociodemographic factors can impact the likelihood of a person consuming tobacco via either method in rural Bangladesh. The prevalence of smoking was found to be higher in males and SLT in females across the lifespan with a declining trend of smoking in males after the age of 60 years. Irrespective of gender, laborious occupation is associated with a higher prevalence of smoking. Psychological distress was associated with smoking in females only. Given that smoking is a major risk factor for cardiovascular disease and cancer, our results add further weight to the necessity for greater emphasis on systemic risk factors in the management of these conditions and to conduct different health programs for the cessation of smoking. This is particularly imperative in rural areas of the community where the level of education, awareness of primary prevention, and access to specialist services are limited and psychological distress among general people is very high.

## Figures and Tables

**Table 1:** Sociodemographic characteristics of the study sample, by gender.

	Number	Female, *N* = 1238 (51%)	Male, *N* = 1187 (49%)	*p*
		Number (%)	Number (%)	
*Age*, *in years*				<0.001
18–39	670	400 (32.3)	270 (22.7)	
40–59	608	269 (21.7)	339 (28.6)	
60–69	883	464 (37.5)	419 (35.3)	
70+	264	105 (8.5)	159 (13.4)	

*Education level*				<0.001
No education	671	382 (30.9)	289 (24.3)	
Primary	946	492 (39.7)	454 (38.2)	
Secondary or higher education	808	364 (29.4)	444 (37.4)	

*Marital status*				<0.001
Married	1937	878 (71.2)	1059 (89.3)	
Widowed	405	333 (27)	72 (6.1)	
Unmarried or never married	78	23 (1.9)	55 (4.6)	

*Occupation*				<0.001
House duties	976	959 (77.5)	17 (1.4)	
Student	40	23 (1.9)	17 (1.4)	
Land owner	217	3 (0.2)	214 (18)	
Daily labours	282	10 (0.8)	272 (22.9)	
Business	220	2 (0.2)	218 (18.4)	
Govt. or nongovt. job	137	13 (1.1)	124 (10.4)	
Retired or unable to work	543	226 (18.3)	317 (26.7)	
Unemployed	10	2 (0.2)	8 (0.7)	

*Socioeconomic status*				0.65
Insufficient money all the time	367	196 (15.8)	171 (14.4)	
Insufficient funds some of the time	781	404 (32.6)	377 (31.8)	
Medium (not good or bad)	1037	516 (41.7)	521 (43.9)	
Sufficient funds most of the time	240	122 (9.9)	118 (9.9)	

*Current health problem*				0.03
No	1063	516 (41.7)	547 (46.1)	
Yes	1362	722 (58.3)	640 (53.9)	
*Number of health problems*				0.006
One or two conditions	763	358 (28.9)	405 (34.1)	
3 or more conditions	1662	880 (71.1)	782 (65.9)	

*Psychological distress*				0.19
Normal	1149	567 (45.8)	582 (49.0)	
Mild	551	296 (23.9)	255 (21.5)	
Moderate	507	254 (20.5)	253 (21.3)	
Severe	218	121 (9.8)	97 (8.2)	

**Table 2:** Smoking status by age and gender in a rural district in Bangladesh.

	Never smoked	Past smoker	Current smoker	SLT use	Tobacco smoking and SLT use
Total	1180 (48.7)	36 (1.5)	621 (25.6)	569 (23.5)	19 (0.8)

*Gender*					
Female	750 (60.6)	0	113 (9.1)	375 (30.3)	0
Male	430 (36.2)	36 (3.0)	508 (42.8)	194 (16.3)	19 (1.6)

*Age group*					
18–39	496 (74)	1 (0.1)	135 (20.1)	37 (5.5)	1 (0.1)
40–59	304 (50)	1 (0.2)	194 (31.9)	102 (16.8)	7 (1.2)
60–69	292 (33.1)	19 (2.2)	238 (27.0)	328 (37.1)	6 (0.7)
70+	88 (33.3)	15 (5.7)	54 (20.5)	102 (38.6)	5 (1.9)

**Table 3:** Prevalence of current or past smoking and the factors associated in the total sample and by gender in a rural district in Bangladesh.

	Never smoker, *N* = 1180 (49%)	Tobacco smoker ever, *N* = 656 (27.1%)		Smokeless tobacco, *N* = 570 (23.5%)	
	*N*	Total		Total	
	*n* (%)	*n* (%)	OR (95% CI)^*∗*^	*n* (%)	OR (95% CI)^*∗*^
*Age group*					
18–39	496 (74.1)	135 (20.2)	1.00 (reference)	38 (5.7)	1.00 (reference)
40–59	304 (50.6)	195 (32.4)	2.36 (1.81, 3.06)	102 (17.0)	4.38 (2.94, 6.53)
60–69	292 (33.3)	257 (29.3)	3.23 (2.51, 4.17)	328 (37.4)	14.7 (10.2, 21.1)
70 and above	88 (34)	69 (26.6)	2.88 (1.99, 4.16)	102 (39.4)	15.1 (9.78, 23.4)

*Gender*					
Females	750 (61)	113 (9.1)	1.0	375 (30.3)	1.0
Males	430 (36.8)	543 (46.5)	8.38 (6.63, 10.6)	195 (16.7)	0.91 (0.74, 1.12)

*Education*					
No education	218 (33)	172 (26.0)	2.71 (1.97, 3.72)	84 (10.4)	1.00
Primary education	408 (43.4)	317 (33.7)	3.47 (2.67, 4.52)	215 (22.9)	2.81 (2.08, 3.80)
Secondary and above education	554 (68.8)	167 (20.7)	1.00	271 (41.0)	3.88 (2.82, 5.36)

*Marital status*					
Married	981 (51.1)	563 (29.4)	1.00	374 (19.5)	1.00
Widowed	137 (33.8)	77 (19.0)	1.54 (1.03, 2.30)	191 (47.2)	1.24 (0.91, 1.68)
Single or unmarried	59 (75.6)	15 (19.2)	0.43 (0.23, 0.79)	4 (5.1)	0.45 (0.15, 1.34)

*Occupation*					
House work	643 (65.9)	74 (7.6)	1.0	259 (26.5)	1.0
Student	39 (97.5)	1 (2.5)	0.32 (0.04, 2.41)	0	—
Land owner	55 (25.7)	123 (57.5)	7.15 (4.04, 12.7)	36 (16.8)	2.02 (1.1, 3.7)
Daily labours	74 (26.9)	173 (62.9)	7.97 (4.65, 13.7)	28 (10.2)	1.05 (0.57, 1.96)
Business	91 (42.3)	108 (50.2)	5.54 (3.18, 9.65)	16 (7.4)	0.64 (0.32, 1.26)
Government or nongovernment job	81 (59.6)	41 (30.1)	2.72 (1.49, 4.96)	14 (10.3)	0.79 (0.39, 1.57)
Retired or unable to work	190 (35.2)	133 (24.6)	1.36 (0.83, 2.21)	217 (40.2)	0.78 (0.54, 1.13)
Unemployed	7 (70)	3 (30)	3.86 (0.93, 16.1)	0	—

*Socioeconomic status*					
Insufficient funds all the time	141 (39.1)	134 (37.1)	1.67 (1.06, 2.64)	86 (23.8)	1.05 (0.64, 1.72)
Insufficient funds some of the time	341 (43.9)	200 (25.8)	0.96 (0.63, 1.44)	235 (30.3)	1.41 (0.92, 2.16)
Medium (not good or bad)	562 (54.6)	262 (25.5)	0.92 (0.62, 1.34)	205 (19.9)	1.01 (0.67, 1.53)
Sufficient funds most of the time	136 (56.7)	60 (25)	1.00	44 (18.3)	1.00

*Health condition*					
No	626 (59.3)	299 (28.3)	1.00	131 (12.4)	1.00
Yes	554 (41)	357 (26.4)	1.0 (0.78, 1.28)	439 (32.5)	1.79 (1.39, 2.32)

*Number of health conditions*					
1-2 conditions	395 (52.9)	167 (22.4)	1.00	185 (24.8)	1.0
3 or more conditions	785 (47.3)	489 (29.5)	1.46 (1.14, 1.88)	385 (23.2)	0.54 (0.42, 0.70)

*Psychological distress*					
Not distressed	617 (54.4)	281 (24.8)	1.0	236 (20.8)	1.0
Mild	277 (50.5)	138 (25.2)	1.26 (0.95, 1.67)	133 (24.3)	1.27 (0.95, 1.69)
Moderate	218 (43.1)	165 (32.6)	1.67 (1.25, 2.23)	123 (24.3)	1.05 (0.77, 1.42)
Severe	68 (31.2)	72 (33.0)	1.93 (1.25, 2.99)	78 (35.8)	0.98 (0.65, 1.47)

^*∗*^OR (95% CI) adjusted for age, gender, level of education, occupation, and marital status.

**Table 4:** Prevalence of smokeless tobacco use and the factors associated in total participants and by gender in a rural district in Bangladesh.

	Never smoker		Tobacco smoker ever				Smokeless tobacco			
	Females	Males	Females, *N* = 113 (9.1%)		Males, *N* = 543 (46.5%)		Females, *n* = 375 (30.3%)		Males, *n* = 195 (16.7)	
	*N* (%)	*N* (%)	*n* (%)	OR (95% CI)^*∗*^	*n* (%)	OR (95% CI)^*∗*^	*n* (%)	OR(95% CI)^*∗*^	*n* (%)	OR (95% CI)^*∗*^
*Age group*										
18–39	364 (91)	132 (49)	8 (2.0)	1.0 (reference)	127 (47)	1.0 (reference)	28 (7.0)	1.0 (reference)	10 (3.7)	1.0 (reference)
40–59	184 (68)	120 (36)	16 (5.9)	3.96 (1.66, 9.4)	179 (54)	1.55 (1.11, 2.17)	69 (26)	4.9 (3.04, 7.8)	33 (9.9)	3.63 (1.72, 7.7)
60–69	172 (37)	120 (29)	76 (16)	20.1 (9.49, 43)	181 (44)	1.57 (1.12, 2.19)	216 (47)	16.3 (10.6, 25)	112 (27.1)	12.32 (6.2, 24)
70 and above	30 (29)	58 (38)	13 (12)	19.7 (7.58, 51)	56 (36)	1.00 (0.65, 1.56)	62 (59)	26.9 (15.0, 48)	40 (26.9)	9.1 (4.3, 19)

*Education*										
Secondary and above	320 (88)	234 (53)	37 (9.7)	3.78 (1.57, 9.1)	135 (48)	2.69 (1.87, 3.87)	37 (10)	1.00	47 (10.7)	1.00
Primary level	289 (59)	119 (27)	69 (14)	6.86 (3.03, 16)	248 (55)	3.06 (2.27, 4.13)	134 (27)	2.59 (1.7, 4.0)	81 (18.1)	2.86 (1.83, 4.47)
No education	141 (37)	77 (28)	7 (1.9)	1.00	160 (36)	1.00	204 (53)	4.4 (2.82, 6.9)	67 (24)	2.64 (1.62, 4.28)

*Marital status*										
Married	612 (70)	369 (35)	64 (7.3)	1.00	499 (48)	1.00	202 (23)	1.00	172 (16.5)	1.00
Widowed	115 (35)	22 (31)	49 (15)	1.18 (0.71, 1.96)	28 (39)	0.72 (0.39, 1.33)	169 (51)	1.18 (0.8, 1.7)	22 (30.6)	0.98 (0.50, 1.93)
Single or unmarried	20 (87)	39 (71)	—	—	15 (27)	0.33 (0.17, 0.63)	3 (13)	—	1 (2)	—

*Occupation*										
House work	635 (66)	8 (47)	71 (7.4)	1.0	3 (18)	1.0	253 (26)	1.0	6 (35.3)	1.0
Student	23 (100)	16 (94)	0	—	1 (5.9)	0.26 (0.02, 2.93)	0	—	0	—
Land owner	1 (33)	54 (26)	2 (67)	20 (1.78, 224)	121 (57)	4.82 (1.21, 19.2)	0	—	36 (17.1)	0.95 (0.28, 3.21)
Daily labours	5 (50)	69 (26)	5 (50)	6.66 (1.67, 27)	168 (63)	5.12 (1.3, 20.2)	0	—	28 (10.6)	0.58 (0.17, 1.97)
Business	1 (50)	90 (42)	1 (50)	6.9 (0.35, 136)	107 (50)	3.53 (0.89, 14)	0	—	16 (7.5)	0.29 (0.08, 1.03)
Government or nongovernment job	12 (92)	69 (56)	1 (7.7)	0.80 (0.1, 6.72)	40 (33)	1.92 (0.47, 7.8)	0	—	14 (11.4)	0.39 (0.11, 1.43)
Retired or unable to work	71 (31)	119 (38)	33 (15)	0.82 (0.45, 1.48)	100 (32)	1.21 (0.3, 4.9)	122 (54)	1.21 (0.3, 4.9)	95 (30.3)	0.41 (0.13, 1.38)
Unemployed	2 (100)	5 (63)	0 (0)	—	3 (38)	2.57 (0.36, 18)	0	—	0	—

*Socioeconomic status*										
Insufficient funds all the time	95 (49)	46 (28)	35 (18)	0.76 (0.36, 1.59)	99 (60)	2.37 (1.36, 4.15)	66 (34)	1.18 (0.6, 2.2)	20 (12.1)	0.85 (0.38, 1.87)
Insufficient funds some time	224 (55)	117 (31)	24 (5.9)	0.26 (0.12, 0.53)	176 (47)	1.77 (1.09, 2.88)	156 (39)	1.56 (0.9, 2.8)	79 (21.2)	1.30 (0.69, 2.43)
Medium (not good or bad)	354 (69)	208 (41)	32 (6.2)	0.27 (0.14, 0.52)	230 (45)	1.63 (1.03, 2.57)	130 (25)	1.06 (0.6, 1.9)	75 (14.6)	0.97 (0.54, 1.76)
Sufficient funds mostly	77 (63)	59 (50)	22 (18)	1.00	38 (32)	1.00	23 (19)	1.00	21 (17.8)	1.00

*Health condition*										
No	407 (79)	219 (41)	23 (4.5)	1.00	276 (51)	1.0	86 (17)	1.00	45 (8.3)	1.00
Yes	343 (48)	211 (34)	90 (13)	2.17 (1.28, 3.65)	267 (43)	0.81 (0.60, 1.08)	289 (40)	1.86 (1.4, 2.6)	150 (23.9)	1.80 (1.16, 2.78)

*Health conditions*										
1–2 conditions	226 (63)	169 (43)	12 (3.4)	1.00	155 (40)	1.00	120 (34)	1.0	65 (16.7)	1.0
3 or more conditions	524 (60)	261 (34)	101 (12)	2.33 (1.21, 4.47)	388 (50)	1.38 (1.04, 1.83)	255 (29)	0.45 (0.3, 0.6)	130 (16.7)	0.75 (0.50, 1.12)

*Psychological distress*										
No distressed	389 (69)	228 (40)	26 (4.6)	1.0	255 (45)	1.0	152 (27)	1.0	84 (14.8)	1.0
Mild	186 (63)	91 (36)	27 (9.1)	2.12 (1.67, 3.83)	111 (44)	1.12 (0.80, 1.56)	83 (28)	1.1 (0.76, 1.6)	50 (19.8)	1.60 (1.01, 2.54)
Moderate	136 (54)	82 (33)	37 (15)	2.98 (1.68, 5.28)	128 (51)	1.38 (0.98, 1.94)	81 (32)	1.0 (0.67, 1.5)	42 (16.7)	1.13 (0.70, 1.84)
Severe	39 (32)	29 (30)	23 (19)	3.55 (1.76, 7.2)	49 (51)	1.28 (0.76, 2.16)	59 (49)	1.1 (0.66, 1.9)	19 (19.6)	0.74 (0.37, 1.48)

^*∗*^OR (95% CI) adjusted for age, gender, level of education, occupation, and marital status.

## Data Availability

The data used in SPSS to support the findings of this study are available from the corresponding author upon request.
